# An Improved Two-Stage Planning Method for Multi-UAV Collaborative Inspection of Spatially Non-Uniform Power Grids

**DOI:** 10.3390/s26206420

**Published:** 2026-10-11

**Authors:** Jianjun Ni, Zhihao Xiao, Chengyuan Zha, Yang Gu, Pengfei Shi

**Affiliations:** College of Artificial Intelligence and Automation, Hohai University, Changzhou 213200, China; 241314010034@hhu.edu.cn (Z.X.); zhcy0521@hhu.edu.cn (C.Z.); 20231153@hhu.edu.cn (Y.G.); shipf@hhu.edu.cn (P.S.)

**Keywords:** power grid inspection, multi-UAV system, spectral clustering, deep reinforcement learning

## Abstract

Autonomous inspection of large-scale power transmission grids using multi-UAV systems faces significant challenges, such as the optimal spatial partitioning due to topological heterogeneity, and the individual trajectory planning due to the geometric sensitivity and data scarcity. To address these issues, this paper proposes a two-stage coordinated planning method. In the proposed method, a distribution-aware reweighted discrete balanced spectral clustering (DAR-DBSC) algorithm is designed to dynamically partition the power grid. Through a dynamic feedback optimization mechanism, it improves workload balance while preserving local spatial affinity structure. In addition, an attention-based deep reinforcement learning solver, named spatial symmetric geometric consistency and per-step smooth entropy annealing algorithm (SSGC-PSEA), is proposed for local trajectory generation. In the proposed SSGC-PSEA algorithm, SSGC extracts geometrically invariant features to improve robustness, while PSEA mitigates gradient shocks to ensure training stability. Furthermore, a “Sim-to-Real” curriculum learning strategy is introduced to overcome real-world data scarcity. Extensive experiments on real-world power grid datasets show that the proposed method outperforms the considered baselines in workload balance, optimization accuracy, and cross-scale generalization.

## 1. Introduction

Autonomous inspection of large-scale power transmission grids is critical for ensuring the security and stability of modern energy infrastructure [1,2,3]. The complex, unstructured environments require systematic monitoring for fault detection (such as identifying shattered insulators, corroded tower structures, and loose hardware), routine maintenance, and asset management [4,5,6]. While individual unmanned aerial vehicles (UAVs) excel in localized inspections due to their maneuverability and operational flexibility, they face significant scalability challenges [7]. Finite battery endurance and extensive geographical coverage requirements limit their efficiency, often resulting in inspection blind spots and resource underutilization [8]. Consequently, the growing complexity of power grids demands innovative inspection strategies that ensure both high operational efficiency and robust environmental adaptability.

Recently, multi-UAV collaborative inspection has advanced rapidly, emerging as an effective approach to overcome the limitations of single-UAV systems [9,10,11]. By integrating distributed task allocation and parallel execution, multi-UAV frameworks achieve significant improvements in overall inspection efficiency [12]. Beyond mere operational scaling, these improvements are driven by advances in key capabilities—specifically cooperative path planning, synchronized flight, and multi-agent workload balancing [13]. Collectively, these advancements enhance the operational capacity of UAV swarms in complex environments, laying a solid technical foundation for the autonomous inspection of large-scale transmission grids [14].

Despite this progress, multi-UAV collaborative inspection still faces notable challenges in practical applications [15]. The conflict between complex power grid geometries and rigid allocation mechanisms complicates optimal spatial partitioning [16]. Real-world power transmission networks typically exhibit high topological heterogeneity, encompassing both dense urban substation hubs and sparse linear corridors across mountainous terrain. Conventional spatial partitioning algorithms often fail to simultaneously achieve satisfactory workload balance and preserve local spatial affinity structure [17,18]. Such limitations can fragment local spatial associations, leading to inefficient inspection paths. Furthermore, unbalanced task allocation induces the “straggler effect” during parallel operations, where the overall inspection progress is delayed by a few overloaded UAVs [19].

Regarding fine-grained trajectory planning for individual UAVs, current Neural Combinatorial Optimization (NCO) algorithms face dual bottlenecks in practical deployment: limited generalization and training instability [20]. These models are highly prone to overfitting the absolute coordinates of training data, struggling to maintain the geometric robustness required to handle spatial transformations, such as the rotation or translation of power grid topologies [21]. Moreover, when optimizing within high-dimensional solution spaces, conventional reinforcement learning mechanisms often fail to achieve a smooth transition between exploration and exploitation. This rigid policy scheduling frequently induces gradient oscillations and destabilizes network convergence during training [22,23]. Fundamentally, the inherent scarcity of empirical field data limits the generalization potential of these models for industrial applications. Since acquiring the true spatial distribution characteristics of transmission towers is costly and yields only small sample sizes, training a model from scratch on such limited datasets often leads to overfitting [24].

To address these challenges, this paper proposes an integrated cyber-physical architecture for multi-UAV collaborative inspection. At the core of this framework is the distribution-aware reweighted discrete balanced spectral clustering (DAR-DBSC) algorithm. By integrating adaptive anchor graph construction with dynamic feedback optimization, DAR-DBSC transforms conventional static spatial partitioning into a dynamic balancing process, substantially reducing workload imbalance while preserving local spatial affinity structure. Following this balanced spatial partitioning, we further introduce a deep reinforcement learning architecture—Spatial symmetric geometric consistency and per-step smooth entropy annealing (SSGC-PSEA) algorithm—for individual UAV trajectory generation. To enhance geometric robustness, SSGC-PSEA enforces spatial symmetric consistency constraints to extract geometrically invariant features. Concurrently, it employs a continuous entropy annealing strategy to suppress convergence fluctuations during training. To overcome the scarcity of empirical field data, a two-stage “Sim-to-Real” curriculum learning mechanism is incorporated, improving the model’s generalization and adaptability within the target physical domain.

In summary, the main contributions of this paper are as follows:

(1) A two-stage hierarchical planning model is proposed for multi-UAV wide-area power grid inspections. By coupling macroscopic spatial partitioning with microscopic trajectory optimization, it effectively decouples grid topological complexity and achieves efficient global resource scheduling.
(2) A distribution-aware reweighted discrete balanced spectral clustering (DAR-DBSC) algorithm is designed. Utilizing a closed-loop dynamic feedback mechanism, it improves workload balance among multiple UAVs while preserving local spatial affinity structure.
(3) A geometry-aware deep reinforcement learning solver (SSGC-PSEA) integrated with a “Sim-to-Real” curriculum is proposed for trajectory planning. It overcomes Euclidean anisotropic biases and training gradient shocks, while leveraging synthetic geometric priors to rapidly adapt to real-world grids, effectively resolving industrial data scarcity.

The remainder of this paper is organized as follows. Section 2 presents an overview of the related work concerning this topic. Section 3 details the core methodology and mathematical frameworks of the DAR-DBSC and SSGC-PSEA algorithms. Section 4 introduces the experimental validation results and the performance analysis. Section 5 gives out the ablation study of the proposed two-stage hierarchical planning model. Section 6 discusses the practical application potential and limitations of the proposed model. Finally, Section 7 concludes this paper.

## 2. Related Work

Driven by the demands of multi-UAV collaborative inspection in complex power transmission grid environments, existing research has extensively investigated key technologies, including spatial partitioning, task coordination, and trajectory generation. This section systematically reviews the core literature pertinent to the proposed architecture.

### 2.1. Spatial Partitioning and Task Scheduling for Multi-UAV Systems

For decentralized multi-UAV systems, effective task coordination and spatial partitioning are fundamental prerequisites for efficient operations [25,26]. The core objective of the partitioning phase is to decompose the wide-area inspection space into mutually exclusive and load-balanced sub-regions. Early spatial partitioning strategies predominantly relied on distance-based heuristics, with classic methods such as Voronoi diagrams and conventional K-means clustering segmenting the target domain based on spatial proximity. Subsequent research refined these algorithms by incorporating UAV kinematic constraints and initial energy states into clustering centroids [27]. However, these centroid-driven strategies remain strictly bound to the assumption of isotropic Euclidean distances. When applied to heterogeneous grids characterized by dense substation hubs intertwined with sparse corridors, such mechanisms frequently disrupt the inherent topological connectivity. Consequently, they generate physically infeasible flight blind spots and incur significant cross-cluster scheduling overheads [28].

To accurately characterize complex geometric manifolds in real-world scenarios, algorithms rooted in graph theory and spectral clustering have increasingly been introduced to spatial partitioning tasks [29]. By leveraging affinity matrix construction and low-dimensional feature space mapping, spectral methods can efficiently identify intrinsic non-linear structures [30]. For instance, in autonomous robotics exploration, generalized spectral clustering has enabled effective classification of non-convex obstacles [31]. However, the direct application of standard spectral algorithms to multi-UAV inspection reveals a critical bottleneck: the challenge of achieving satisfactory workload balance. Fundamentally, conventional spectral methods prioritize graph cut minimization in their objective functions, precluding the explicit enforcement of cluster size constraints [32].

To ensure a balanced allocation of operational workloads, balanced clustering algorithms with size constraints have been progressively developed. Among these, Discrete Balanced Spectral Clustering (DBSC) has emerged as an established representative [33]. The DBSC framework unifies continuous relaxation and discrete indicator optimization, employing a global regularization term controlled by a static scalar to suppress unbalanced partitioning. However, in the context of highly heterogeneous grid topologies, the reliance on static scalar penalty mechanisms reveals inherent limitations. The model faces a rigid parameter trade-off: weak penalties fail to overcome the strong spectral connectivity within dense substation areas, yielding oversized clusters; conversely, excessive penalties disrupt the sparse topological structures of linear corridors. This conflict induces cluster fragmentation and triggers the “straggler effect”, where overloaded units hinder the overall inspection efficacy.

To overcome the inherent limitations of the standard DBSC framework, the proposed DAR-DBSC algorithm in this paper reconciles spatial geometric complexity with workload balancing requirements.

### 2.2. Trajectory Generation Based on Neural Combinatorial Optimization

Following spatial partitioning, fine-grained trajectory planning for individual UAVs is typically formulated as a constrained Traveling Salesman Problem (TSP) [34]. Traditional Operations Research (OR) approaches rely on exact solvers (e.g., Concorde) or heuristic algorithms (e.g., LKH, genetic algorithms, ant colony optimization) to search for optimal routes. While capable of yielding high-quality solutions, these methods incur prohibitive computational overhead and exhibit limited scalability, precluding real-time deployment in dynamic multi-UAV systems [35].

Recently, Neural Combinatorial Optimization (NCO) has emerged as a promising alternative to address these bottlenecks, leveraging highly efficient real-time inference capabilities [36]. Early NCO models predominantly relied on Recurrent Neural Networks (RNNs) and Pointer Networks to iteratively generate node sequences [37]. However, the sequential nature of these mechanisms inherently restricted representational capacity. The subsequent introduction of Transformer architectures and attention mechanisms significantly expanded feature extraction capabilities [38]. Notably, the Policy Optimization with Multiple Optima (POMO) model has achieved state-of-the-art performance across various routing tasks by integrating multi-start decoding with shared-baseline reinforcement learning [39].

Despite these algorithmic advancements, the direct application of NCO to power grid inspection still suffers from geometric sensitivity and optimization instability [40]. Current attention mechanisms rely heavily on absolute coordinate encoding, rendering models sensitive to spatial anisotropy. The policy network tends to overfit specific spatial orientations, failing to extract rotation-invariant features from the grid topology. Identical transmission corridors with different rotation angles are frequently misclassified as heterogeneous samples [21]. Separately, Deep Reinforcement Learning (DRL)-based path planning models experience pronounced convergence oscillations during training [41]. Conventional frameworks employ coarse, epoch-based discrete temperature scheduling to balance exploration and exploitation [42]. Such rigid parameter updates induce “gradient shocks”, trapping the network in local optima. Furthermore, the inherent scarcity of empirical grid data exacerbates these generalization bottlenecks. Predominantly trained on uniformly distributed synthetic instances, existing models fail to capture the sharp topological density gradients inherent in physical grids, suffering significant performance degradation during deployment.

To overcome geometric sensitivity, suppress optimization oscillations, and resolve generalization bottlenecks under few-shot conditions in this field, this paper introduces the SSGC-PSEA deep reinforcement learning architecture. Departing from traditional NCO models, this architecture discards the reliance on absolute coordinates and discrete scheduling schemes.

## 3. Methodology

### 3.1. Problem Definition

Multi-UAV cooperative inspection fundamentally relies on partitioning a widely distributed set of transmission towers into mutually exclusive sub-regions. Let $P=\{p_{1},p_{2},\ldots ,p_{n}\}$ denote the set of *n* inspection nodes in the original geodetic coordinate system. Given the spatial span of long-distance power lines, calculating Euclidean distances directly in a spherical space introduces significant geometric distortion, which subsequently degrades the construction accuracy of spectral embedding features.

To mitigate this, the geodetic coordinates are transformed into a local Cartesian coordinate system via equirectangular projection. Geometry-preserving scaling is then applied to the projected physical coordinates to eliminate dimensional discrepancies. This process constructs a normalized two-dimensional input dataset, $X=\left\{x_{1},x_{2},\ldots ,x_{n}\right\}\in R^{n\times 2}$, while strictly preserving the relative topology of the network. For an inspection fleet comprising *c* homogeneous UAVs, this spatial partitioning task is mathematically formulated as finding a binary indicator matrix $Y\in {\left\{0,1\right\}}^{n\times c}$ to precisely assign each inspection node to a specific UAV.

The partitioning process is governed by two core constraints: mutual exclusivity and workload balance. Mutual exclusivity ensures complete, non-redundant task coverage by assigning each node to exactly one UAV, algebraically expressed as $Y1_{c}=1_{n}$ (where $1_{c}$ and $1_{n}$ denote all-ones vectors of dimensions *c* and *n*). Furthermore, to guarantee synchronized flight cycles, the multi-UAV system requires highly consistent operational workloads, formally defined as $1_{n}^{T}Y\approx \frac{n}{c}1_{c}^{T}$. While binary discrete solutions inherently satisfy mutual exclusivity, traditional spectral clustering often struggles to achieve a satisfactory workload balance on spatially non-uniform data, frequently yielding highly uneven task distributions.

### 3.2. Distribution-Aware Reweighted Discrete Balanced Spectral Clustering

To address the inherent conflict between non-uniform spatial distributions and workload balancing in multi-UAV power grid inspections, a distribution-aware reweighted discrete balanced spectral clustering (DAR-DBSC) algorithm is proposed. By integrating adaptive graph construction with a dynamic feedback-driven reweighting strategy, traditional static spatial partitioning is transformed into a dynamic balancing optimization.

Solving this combinatorial optimization problem relies on a spectral relaxation strategy. To reduce the time complexity of graph construction and eigendecomposition, *m* anchors are utilized to approximate the original network structure. Let $B\in R^{n\times m}$ denote the normalized affinity matrix between the nodes and the anchors. Based on this low-rank bipartite graph approximation, the topology of the power grid is mapped into a Laplacian matrix $L=I-BB^{T}$. Under the orthogonality constraint $F^{T}F=I$, classical spectral clustering algorithms typically compute the continuous spectral embedding $F\in R^{n\times c}$ by minimizing the Laplacian trace term $\mathrm{Tr}(F^{T}LF)$, which is equivalent to maximizing the affinity trace $\mathrm{Tr}(F^{T}BB^{T}F)$.

However, the directly derived continuous feature subspace generally fails to align with the discrete and balanced structures required by the indicator matrix *Y*. To bridge the continuous geometric manifold and the discrete operational constraints, the following joint optimization objective is formulated:

(1) $$\max_{F,R,Y}J=\mathrm{Tr}\left(F^{T}BB^{T}F\right)+\gamma \mathrm{Tr}\left(R^{T}F^{T}Y\right)$$

where the optimization variables must satisfy the orthogonality constraint $F^{T}F=I$; $R\in R^{c\times c}$ is an orthogonal rotation matrix satisfying $R^{T}R=I$; and the indicator matrix *Y* strictly follows the discrete assignment and mutual exclusivity constraints, i.e., $Y\in {\left\{0,1\right\}}^{n\times c}$ and $Y1_{c}=1_{n}$. In the aforementioned objective function, the first term aims to preserve the local connectivity encoded by the constructed affinity graph, which is equivalent to minimizing the graph cut. The second term, modulated by the parameter γ, acts as a regularization term forcing the continuous spectral embedding *F* to approximate the discrete configuration $YR^{T}$.

In spatially non-uniform power grids, a static global penalty γ often fails under sharp density gradients: small values cannot enforce load balancing in dense hubs, whereas large values fracture sparse corridors. Consequently, the static γ is replaced by a dynamic, feedback-driven reweighting mechanism, as derived below.

#### 3.2.1. Distribution-Aware Anchor Graph Construction

Conventional spectral clustering incurs an $O(n^{2})$ complexity, restricting its application in large-scale grids. To enhance scalability, a landmark-based approximation is employed: K-means clustering extracts *m* anchors $U=\{u_{1},u_{2},\ldots ,u_{m}\}$ (m≪n) from the projected dataset *X*. Constructing a bipartite graph between the original nodes and these anchors yields the affinity matrix *B*. This low-rank approximation transforms dense graph construction into a linear sparse mapping, drastically reducing computational overhead while preserving local spatial affinity structure.

Traditional Gaussian kernels with a static bandwidth σ fail in spatially non-uniform grids: small σ values disconnect sparse corridors, while large ones generate spurious edges in dense hubs. Thus, a distribution-aware similarity metric with an adaptive bandwidth is designed. For each node $x_{i}$, a local scale parameter $\sigma_{i}$ is defined by its distance to the *k*-th nearest anchor:

(2) $$\sigma_{i}=\max\left(\mathrm{dist}\left(x_{i},u_{\left(k\right)}\right),\epsilon \right)$$

where $u_{\left(k\right)}$ denotes the *k*-th nearest anchor, and ϵ is a sufficiently small constant to prevent numerical singularity.

This dynamically shrinks $\sigma_{i}$ in dense hubs for higher resolution and expands it in sparse corridors to maintain connectivity. Thus, the affinity $z_{ij}$ between node $x_{i}$ and anchor $u_{j}$ is computed via the following truncated adaptive kernel:

(3) $$z_{ij}=\left\{\begin{array}{cc} \exp\left(-\frac{∥x_{i}-u_{j}{∥}^{2}}{2\sigma_{i}^{2}}\right), & \mathrm{if}\;u_{j}\in N_{s}\left(x_{i}\right)\\ 0, & \mathrm{otherwise} \end{array}\right.$$

where $N_{s}\left(x_{i}\right)$ denotes the *s* nearest anchors to $x_{i}$. This truncation yields a sparse association matrix $Z\in R^{n\times m}$, filtering distant noise to preserve the local manifold geometry.

Normalizing the sparse matrix *Z* by column degree yields the low-rank affinity matrix *B*, computed as:

(4) $$B=Z\Lambda^{-1/2}$$

where $\Lambda \in R^{m\times m}$ is a diagonal degree matrix with $\Lambda_{jj}=\sum_{i=1}^{n}z_{ij}$. This normalization balances local density variations across anchors, ensuring probabilistic transition properties for the bipartite graph.

Based on spectral graph theory, the global similarity matrix is implicitly formulated as $W=BB^{T}$, yielding the Laplacian $L=I-BB^{T}$. This low-rank decomposition bypasses the explicit construction of a dense global graph. Consequently, alternating matrix multiplication reduces the feature extraction complexity from $O(n^{3})$ to O(nmc) (where *c* is the cluster count), achieving linear scalability with respect to node count *n* for large-scale grids.

#### 3.2.2. Dynamic Feedback-Driven Optimization Mechanism

Conventional static penalties (γ) struggle to balance workloads across spatially non-uniform grids. To address this, a dynamic feedback mechanism based on closed-loop control is introduced. At iteration *t*, the static γ is replaced by a time-varying weight matrix $\Omega^{\left(t\right)}=\mathrm{diag}\left(w_{1}^{\left(t\right)},\ldots ,w_{c}^{\left(t\right)}\right)$, reformulating the joint objective as:

(5) $$J\left(F,R,Y\right)=\mathrm{Tr}\left(F^{T}BB^{T}F\right)+\mathrm{Tr}\left(R^{T}F^{T}Y\Omega^{\left(t\right)}\right)$$

where the adaptive regularization matrix $\Omega^{\left(t\right)}$ adjusts sub-region penalties based on current workload deviations, guiding the continuous spectral embedding toward a discrete partition with reduced workload imbalance.

The joint model is solved via alternating maximization. To prevent numerical oscillations from unstable initial embeddings, a warm-up mechanism is employed: a static baseline penalty aligns the subspace during early iterations ($t\leq T_{\mathrm{warm}}$) before activating the dynamic feedback loop.

At iteration *t*, based on the previous cluster size $n_{j}^{\left(t-1\right)}$, the balancing weights are updated via a nonlinear negative feedback rule:

(6) $$w_{\mathrm{new},j}^{\left(t\right)}=\gamma_{\mathrm{base}}\cdot {\left(\frac{n/c}{n_{j}^{\left(t-1\right)}}\right)}^{\alpha }$$

where $\gamma_{\mathrm{base}}$ is the baseline regularization intensity, and the exponent α adjusts the sensitivity of the feedback response.

Following proportional control, $w_{j}$ for underloaded sub-regions ($n_{j}^{\left(t-1\right)}<n/c$) is nonlinearly amplified to strengthen the regularization $\mathrm{Tr}(R^{T}F^{T}Y\Omega^{\left(t\right)})$, attracting more nodes. Conversely, for overloaded regions, $w_{j}$ is attenuated, enabling the topology-preserving term $\mathrm{Tr}(F^{T}BB^{T}F)$ to dominate and prune marginal nodes. To suppress numerical oscillations during optimization, a momentum smoothing strategy updates the newly computed weight vector $w_{\mathrm{new}}^{\left(t\right)}={\left[w_{\mathrm{new},1}^{\left(t\right)},\ldots ,w_{\mathrm{new},c}^{\left(t\right)}\right]}^{T}$:

(7) $${\tilde{w}}^{\left(t\right)}=\beta {\tilde{w}}^{\left(t-1\right)}+\left(1-\beta \right)w_{\mathrm{new}}^{\left(t\right)}$$

where β is the momentum coefficient used to smooth the weight updates and mitigate numerical oscillations. To prevent weight divergence under extremely unbalanced topologies, the updated weights are strictly bounded within $\left[\gamma_{\min},\gamma_{\max}\right]$.

With the dynamic weights determined, an alternating optimization strategy is employed to update {F,R,Y}. Fixing *R* and *Y*, the update of *F* maximizes $\mathrm{Tr}\left(F^{T}BB^{T}F\right)+\mathrm{Tr}\left(F^{T}G\right)$ subject to $F^{T}F=I$, where $G=Y\Omega^{\left(t\right)}R^{T}$. To solve this Stiefel manifold problem efficiently without traditional eigenvalue decomposition, the Generalized Power Iteration (GPI) method is utilized [43]. The closed-form update relies on the polar decomposition of the gradient matrix $M=2BB^{T}F+G$, yielding $F\leftarrow U_{m}V_{m}^{T}$, where $U_{m}\Sigma_{m}V_{m}^{T}$ is the SVD of *M*.

Fixing *F* and *Y*, the update of *R* optimally aligns the continuous spectral features with the weighted discrete indicator space. This is formulated as a standard Orthogonal Procrustes problem:

(8) $$\max_{R^{T}R=I}\mathrm{Tr}\left(R^{T}F^{T}Y\Omega^{\left(t\right)}\right)$$

which yields the optimal closed-form solution $R=U_{n}V_{n}^{T}$, where $U_{n}\Sigma_{n}V_{n}^{T}$ represents the SVD of the matrix $F^{T}Y\Omega^{\left(t\right)}$.

Fixing *F* and *R*, the update of *Y* maximizes the weighted alignment $\mathrm{Tr}(Y\Omega^{\left(t\right)}R^{T}F^{T})$. Let $P=FR\in R^{n\times c}$ denote the rotated spectral embedding. Constrained by one-hot mutually exclusive assignments, this global optimization decouples into *n* independent subproblems. For each node *i*, the optimal assignment is determined greedily: $y_{ij}=1$ if $j=\arg\;\max_{k}\left(w_{k}^{\left(t\right)}P_{ik}\right)$. Unlike conventional spectral clustering, this mechanism directly outputs discrete labels while incorporating workload-aware reweighting during optimization, circumventing heuristic post-processing (e.g., K-means).

Through the closed-loop alternating optimization of $\Omega^{\left(t\right)}$ and the partitioning parameters, DAR-DBSC produces a near-optimal partition.

Based on the iterative update rules derived previously, the complete implementation of the DAR-DBSC algorithm is summarized in Algorithm 1.
**Algorithm 1** DAR-DBSC Algorithm  1:**Input:** Node coordinates $X\in R^{n\times 2}$, hyperparameters $\left\{c,m,\gamma_{\mathrm{base}},\alpha ,\beta ,T_{\mathrm{start}},T_{\max}\right\}$  2:**Output:** Discrete cluster assignments $Y\in {\left\{0,1\right\}}^{n\times c}$  3:Extract *m* anchors via K-means and construct adaptive bipartite graph *B*;  4:Initialize F,R,Y, and $w^{\left(0\right)}\leftarrow \gamma_{\mathrm{base}}1_{c}$;  5:**for** t=1 **to** $T_{\max}$ **do**  6: Update continuous embedding *F*;  7: Update orthogonal rotation matrix *R*;  8: Update discrete indicator matrix *Y*;  9: **if** $t\geq T_{\mathrm{start}}$ **then**10:  Compute cluster sizes $n_{j}^{\left(t-1\right)}$ from *Y*;11:  Compute $w_{\mathrm{new}}^{\left(t\right)}$ using $n_{j}^{\left(t-1\right)}$ and balance power α;12:  $w^{\left(t\right)}\leftarrow \beta w^{\left(t-1\right)}+\left(1-\beta \right)w_{\mathrm{new}}^{\left(t\right)}$;13: **else**14:  $w^{\left(t\right)}\leftarrow w^{\left(t-1\right)}$;15: **end if**16: **if** convergence criterion is met **then**17:  **break**;18: **end if**19:**end for**20:**return** *Y*

**Theorem** **1.** *For a fixed weight matrix* Ω*, the inner alternating optimization over F, R, and Y in DAR-DBSC produces a non-decreasing sequence of the objective $J_{\Omega }\left(F,R,Y\right)$. Since the feasible set is bounded, the objective sequence converges.*

**Proof.**  For a fixed weight matrix Ω, the joint optimization is decoupled into three subproblems. The following demonstrates that each inner update monotonically increases $J_{\Omega }$.

Fixing *R* and *Y* for the fixed matrix Ω, the update of *F* maximizes $\mathrm{Tr}\left(F^{T}BB^{T}F\right)+\mathrm{Tr}\left(F^{T}G\right)$ subject to $F^{T}F=I$. The Generalized Power Iteration (GPI) algorithm guarantees a monotonic increase in the objective, satisfying:

(9) $$J_{\Omega }\left(F^{\left(t\right)},R^{\left(t-1\right)},Y^{\left(t-1\right)}\right)\geq J_{\Omega }\left(F^{\left(t-1\right)},R^{\left(t-1\right)},Y^{\left(t-1\right)}\right)$$

Fixing *F* and *Y* for the fixed matrix Ω, the update of *R* reduces to an Orthogonal Procrustes Problem maximizing $\mathrm{Tr}(R^{T}F^{T}Y\Omega )$. The SVD-based closed-form solution $R=U_{n}V_{n}^{T}$ is globally optimal for this subproblem, yielding:

(10) $$J_{\Omega }\left(F^{\left(t\right)},R^{\left(t\right)},Y^{\left(t-1\right)}\right)\geq J_{\Omega }\left(F^{\left(t\right)},R^{\left(t-1\right)},Y^{\left(t-1\right)}\right)$$

Fixing *F* and *R* for the fixed matrix Ω, the update of *Y* maximizes $\mathrm{Tr}(Y\Omega R^{T}F^{T})$. Constrained by independent one-hot assignments, greedily selecting the maximum weighted score $w_{k}P_{ik}$ for each node yields an exact discrete solution, guaranteeing:

(11) $$J_{\Omega }\left(F^{\left(t\right)},R^{\left(t\right)},Y^{\left(t\right)}\right)\geq J_{\Omega }\left(F^{\left(t\right)},R^{\left(t\right)},Y^{\left(t-1\right)}\right)$$

Thus, for a fixed Ω, $J_{\Omega }$ is monotonically non-decreasing during the inner alternating optimization. Since *F* and *R* reside in bounded orthogonal spaces and *Y* belongs to a finite discrete space, $J_{\Omega }$ is bounded above. Therefore, $J_{\Omega }$ converges to a finite limit for fixed Ω. When $\Omega^{\left(t\right)}$ is updated by the external feedback loop, the objective function changes between outer iterations; hence, global monotonicity of the complete dynamic procedure is not claimed here. The momentum-smoothed feedback update is introduced to mitigate oscillations. □

### 3.3. Geometry-Aware Deep Reinforcement Learning for Trajectory Generation

Building upon the balanced inspection sub-regions generated by the DAR-DBSC algorithm, local trajectory planning for individual UAVs is formulated as a Traveling Salesman Problem (TSP). To efficiently solve this under complex topological constraints, a geometry-aware deep reinforcement learning architecture, termed SSGC-PSEA, is proposed. This framework incorporates a spatial symmetry-driven geometric consistency (SSGC) module to enhance spatial robustness, a progressively smoothed entropy annealing (PSEA) strategy to stabilize training, and a sim-to-real curriculum learning mechanism to overcome real-world data scarcity.

#### 3.3.1. Local Routing Formulation and RL Baseline

To decouple the wide-area inspection task into solvable local subtasks, the spatial partitioning results generated by the DAR-DBSC algorithm are utilized as local Traveling Salesman Problem (TSP) instances. Consequently, the local trajectory generation is formulated as a Markov Decision Process (MDP). Given the high variance issue of the policy gradient in traditional algorithms, a parallel trajectory sampling and reinforced baseline mechanism based on Policy Optimization with Multiple Optima (POMO) is incorporated as the reinforcement learning baseline [39]. Specifically, a standard attention-based Transformer encoder–decoder architecture is employed to parameterize the policy network $p_{\theta }$. By rolling out multiple trajectories in parallel from different starting nodes within an instance and utilizing the empirical mean reward as a shared baseline, the model effectively computes the policy gradient and minimizes the core reinforcement learning loss $L_{rl}$.

#### 3.3.2. Spatial Symmetry-Driven Geometric Consistency Framework

To address the limitation that conventional attention models are prone to overfitting the absolute node coordinates (x,y) in Euclidean space, a self-supervised geometric consistency (SSGC) learning mechanism based on spatial symmetry is proposed. This mechanism guides the parameterized policy model $p_{\theta }$ to extract relative manifold features with rotation and translation invariance. Specifically, for any input cluster instance $C_{k}$, two sets of spatially symmetric representations are simultaneously constructed:

(1) Original View ($V_{\mathrm{orig}}$): Retains the original absolute coordinate input, i.e., $V_{\mathrm{orig}}=C_{k}$.
(2) Augmented View ($V_{\mathrm{aug}}$): Generates the spatially augmented input $V_{\mathrm{aug}}=T\left(C_{k}\right)$ via a geometric transformation operator T(·), where the operator T encompasses random rotation R(θ), horizontal flipping $F_{h}$, and vertical flipping $F_{v}$.

To enforce the spatial equivalence of the parameterized policy $p_{\theta }$, the two sets of symmetric inputs are fed into the shared-parameter model. Based on the principle of topological isomorphism, geometric transformations of absolute node coordinates should not alter the optimal traversal sequence. Thus, a symmetric Kullback-Leibler (KL) divergence is defined as the consistency loss $L_{\mathrm{cons}}$ to penalize policy prediction deviations across spatial transformations:

(12) $$\begin{array}{ccc} L_{\mathrm{cons}} & = & \frac{1}{2}\left(D_{\mathrm{KL}}\left(p_{\theta }\left(\cdot |C_{k}\right)∥p_{\theta }\left(\cdot |{\tilde{C}}_{k}\right)\right)+D_{\mathrm{KL}}\left(p_{\theta }\left(\cdot |{\tilde{C}}_{k}\right)∥p_{\theta }\left(\cdot |C_{k}\right)\right)\right) \end{array}$$

where ${\tilde{C}}_{k}$ represents the spatially augmented view, and $p_{\theta }\left(\cdot |\cdot \right)$ denotes the conditional probability distribution output by the policy model. During joint optimization, to synergize topological optimization with geometric invariance, the consistency loss $L_{\mathrm{cons}}$ is dynamically coupled with the policy loss $L_{rl}$, yielding the total loss $L_{\mathrm{total}}$:

(13) $$L_{\mathrm{total}}=L_{rl}+\beta L_{\mathrm{cons}}$$

where β is a scalar weighting the consistency penalty. Minimizing $L_{\mathrm{total}}$ injects a geometric equivalence prior into the parameter space while maximizing the advantage reward for the shortest physical path. This self-supervised regularization improves sample efficiency and enhances generalization robustness across unseen heterogeneous topologies (e.g., sparse corridors and complex hubs).

#### 3.3.3. Per-Step Smooth Entropy Annealing (PSEA)

In neural combinatorial optimization, conventional static temperature settings can induce severe policy gradient oscillations. During the rollout of each TSP route, a per-step smooth entropy annealing (PSEA) strategy updates the temperature at every decoding step t∈{0,…,T−1}, where *T* denotes the number of decoding decisions:

(14) $$\tau_{t}=\tau_{\max}{\left(\frac{\tau_{\min}}{\tau_{\max}}\right)}^{\frac{t}{T-1}}$$

where $\tau_{\max}$ and $\tau_{\min}$ are the initial and final temperatures, respectively. The temperature schedule is restarted for each newly generated route.

To ensure a valid non-repetitive TSP tour, a standard dynamic mask $M_{t}$ assigns −∞ to visited nodes. Thus, the action probability $\pi_{t}$ for selecting candidate node *j* given the current state $s_{t}$ at decoding step *t* is formulated as:

(15) $$p_{\theta }\left(\pi_{t}=j|s_{t}\right)=\mathrm{Softmax}\left(\frac{q_{t}k_{j}^{T}}{\tau_{t}\cdot \sqrt{d_{k}}}+M_{t,j}\right)$$

where $q_{t}$ and $k_{j}$ are the attention query and key vectors, respectively, and $d_{k}$ is the feature dimension. At early decoding steps, a higher temperature encourages exploration among candidate nodes. As a route is progressively constructed, the temperature decreases and sharpens the action distribution. This smooth schedule avoids abrupt changes between successive decoding decisions.

#### 3.3.4. Sim-to-Real Domain-Adaptive Curriculum Learning

In industrial deployments, DRL often suffers from data scarcity due to the high acquisition costs of real transmission tower topologies. Training from scratch on such limited datasets exacerbates overfitting. To address this, a two-stage sim-to-real curriculum learning mechanism is introduced to decouple general topological reasoning from domain-specific adaptation.

Stage 1: General pre-training. The model is initially pre-trained on large-scale synthetic instances with uniform distributions by minimizing the joint loss $L_{\mathrm{total}}$. Since uniform distributions provide diverse topological morphologies, this stage guides the SSGC-PSEA framework to extract fundamental geometric rules and long-range dependencies essential for optimal closed-loop tours.

Stage 2: Domain-specific fine-tuning. Initialized with the pre-trained weights, the model is fine-tuned on target domain samples from real-world power transmission networks. Each target-domain sample is constructed by randomly selecting a center node from the real-world grid-coordinate data and extracting its 100 nearest inspection nodes; the resulting local instance is then normalized before fine-tuning. To adapt to the sharp density gradients of physical grids (i.e., interwoven dense hubs and sparse corridors), the learning rate is strictly decayed by an order of magnitude relative to the pre-training phase. Furthermore, a multi-start sampling strategy coupled with the PSEA mechanism is integrated during the decoding phase. This configuration efficiently aligns the policy distribution with the target domain features within a minimal number of epochs, ensuring smooth transfer while preventing catastrophic forgetting.

By employing this curriculum learning strategy, the model first extracts generalizable topological priors from abundant synthetic datasets. Subsequently, it rapidly aligns these learned priors with the heterogeneous spatial distributions inherent in real-world power grids. This paradigm effectively bridges the sim-to-real gap, overcoming the critical bottleneck of domain adaptation under industrial data scarcity. The training procedure of the SSGC-PSEA architecture is summarized in Algorithm 2.
**Algorithm 2** Training Procedure of the SSGC-PSEA Architecture  1:**Input:** Training dataset D, hyperparameters $\left\{E_{\max},B,\eta ,\tau_{\max},\tau_{\min},\beta \right\}$  2:**Output:** Optimized policy parameters $\theta^{*}$  3:Initialize parameters θ;  4:**for** e=1 **to** $E_{\max}$ **do**  5: **for each** batch $C_{k}∼D$ **do**  6:  $V_{\mathrm{orig}}\leftarrow C_{k},\;V_{\mathrm{aug}}\leftarrow T\left(C_{k}\right)$;  7:  **for** $V\in \{V_{\mathrm{orig}},V_{\mathrm{aug}}\}$ **do**  8:   Rollout *N* trajectories, updating $\tau_{t}$ at each decoding step *t*, to obtain rewards   ${\left\{R\left(\pi^{\left(i\right)}\right)\right\}}_{i=1}^{N}$;  9:   Compute baseline $b\leftarrow \frac{1}{N}\sum_{i=1}^{N}R\left(\pi^{\left(i\right)}\right)$;10:  **end for**11:  Compute RL loss $L_{rl}$ via REINFORCE;12:  Compute consistency loss $L_{\mathrm{cons}}$ between $V_{\mathrm{orig}}$ and $V_{\mathrm{aug}}$;13:  Update $\theta \leftarrow \theta -\eta \nabla_{\theta }\left(L_{rl}+\beta L_{\mathrm{cons}}\right)$;14: **end for**15:**end for**16:**return **$\theta^{*}$

## 4. Experiments and Analysis

To evaluate the overall performance of the proposed two-stage coordination framework, systematic experiments are conducted on real-world transmission network datasets. The experimental setup details the physical characteristics of the datasets, the selected baseline algorithms, and the evaluation metrics. The performance analysis focuses on two primary aspects: first, a quantitative assessment of the load-balancing capabilities of the DAR-DBSC algorithm during the spatial partitioning phase; and second, an in-depth analysis of the global optimization performance and computational efficiency of the SSGC-PSEA architecture when navigating complex, heterogeneous topologies. The spatial partitioning and trajectory-planning experiments use the same real-world grid-coordinate data at different scales.

### 4.1. Experimental Setup

To verify the adaptability of the proposed algorithm under spatially non-uniform topologies, three representative experimental scenarios are constructed based on the physical characteristics of real-world transmission grids. The urban region scenario represents a high-density, complex mesh topology. The mountain corridor scenario is predominantly characterized by sparse, chain-like structures, designed to evaluate the algorithm’s capability to maintain long-range connectivity. The energy base scenario simulates the pronounced spatial density gradients between power generation hubs and outgoing backbone networks, serving to validate the algorithm’s effectiveness in handling highly uneven distribution patterns.

For spatial partitioning, each scenario retains a large regional grid. The number of clusters is set to c=[N/100], such that each resulting subregion contains approximately, rather than exactly, 100 nodes. In contrast, the trajectory-planning model is trained and evaluated using TSP-100 instances constructed by selecting exactly 100 nodes from the real-world grid-coordinate data. The model was pre-trained on 160,000 synthetic TSP-100 instances and fine-tuned on 20,000 real-world TSP-100 instances. The test set contains 100 topology-disjoint real-world TSP-100 instances per scenario (300 in total), sampled separately from the fine-tuning data.

For the spatial partitioning phase, the experiments benchmark the DAR-DBSC algorithm against three representative baseline methods: K-Means [44], which partitions based on isotropic Euclidean distance metrics; standard Spectral Clustering (SC) [45], which lacks explicit load-balancing constraints; and Discrete Balanced Spectral Clustering (DBSC) [33], a scalable spectral method reliant on a static regularization parameter. In terms of evaluation metrics, the Balance Standard Deviation ($\sigma_{B}$) and Load Range ($R_{L}$) are employed to quantify the workload balancing among the multi-UAV fleet, which reflects the node-count variation among the subregions. The Silhouette Coefficient (*S*) is utilized to assess the geometric compactness and spatial coherence of the generated clusters. Additionally, Execution Time (*T*) is recorded to compare the computational efficiency of each algorithm across large-scale grid scenarios.

For the trajectory generation phase, building upon the validated effectiveness of the spatial partitioning, the global planning performance of the SSGC-PSEA architecture is systematically evaluated. Crucially, all subsequent evaluations are conducted using the model optimized through the domain-specific fine-tuning stage. This fine-tuned model is benchmarked against the LKH-3 heuristic solver [46] and state-of-the-art Neural Combinatorial Optimization (NCO) baselines to assess solution accuracy and inference efficiency. Additionally, comprehensive experiments are conducted to validate the individual contributions of the SSGC and PSEA mechanisms, examine geometric robustness against spatial transformations, and verify zero-shot extrapolation capability on unseen graph sizes. Finally, trajectory visualizations are provided to demonstrate the architecture’s optimization capabilities across complex physical topologies.

The proposed algorithms are implemented in Python 3.11.15 using the PyTorch 2.7.1 deep learning framework. The testing hardware platform is equipped with an Intel Core i7-10750H CPU (2.60 GHz) and an NVIDIA GeForce RTX 2060 GPU. Performance evaluations on this consumer-grade mobile workstation demonstrate that both the DAR-DBSC and SSGC-PSEA architectures can operate efficiently within compute-constrained execution environments. This indicates their computational feasibility for edge inference on portable UAV ground control stations under the considered static planning setting.

The detailed hyperparameter configurations for the spatial partitioning algorithm (DAR-DBSC) and the trajectory generation neural solver (SSGC-PSEA) are summarized in Table 1 and Table 2, respectively.

### 4.2. Comparative Analysis of Spatial Partitioning

#### 4.2.1. Quantitative Analysis

Table 3 presents the quantitative evaluation results across the three typical scenarios. The proposed DAR-DBSC method outperforms the compared baseline algorithms in task load-balancing metrics. For instance, in the mountain scenario characterized by sparse chain-like corridors, the balance standard deviation ($\sigma_{B}$) of the proposed method is drastically reduced to 3.02, which is nearly an order of magnitude lower than those of K-Means (30.49) and standard Spectral Clustering (38.01). Concurrently, the load range ($R_{L}$) is reduced to 9, demonstrating the effectiveness of the introduced mechanism in minimizing multi-UAV task allocation disparities. Across both the urban city and energy hub scenarios, DAR-DBSC consistently maintains low $\sigma_{B}$ and $R_{L}$ values, effectively alleviating the “long-tail” allocation phenomenon common in traditional graph partitioning.

Computationally, the dynamic feedback mechanism introduces a marginal increase in execution time (approximately 1.2 s) compared to non-iterative algorithms. However, the overhead remains within the same order of magnitude, fulfilling the requirements for online deployment in large-scale grids. Furthermore, while the Silhouette Coefficient (*S*) of DAR-DBSC is slightly lower than K-Means in certain scenarios, it remains highly competitive (e.g., 0.4221 in the mountain scenario). Ultimately, the proposed model effectively preserves intra-cluster geometric compactness while achieving substantially improved workload balance.

#### 4.2.2. Qualitative Analysis

**Figure 1:**
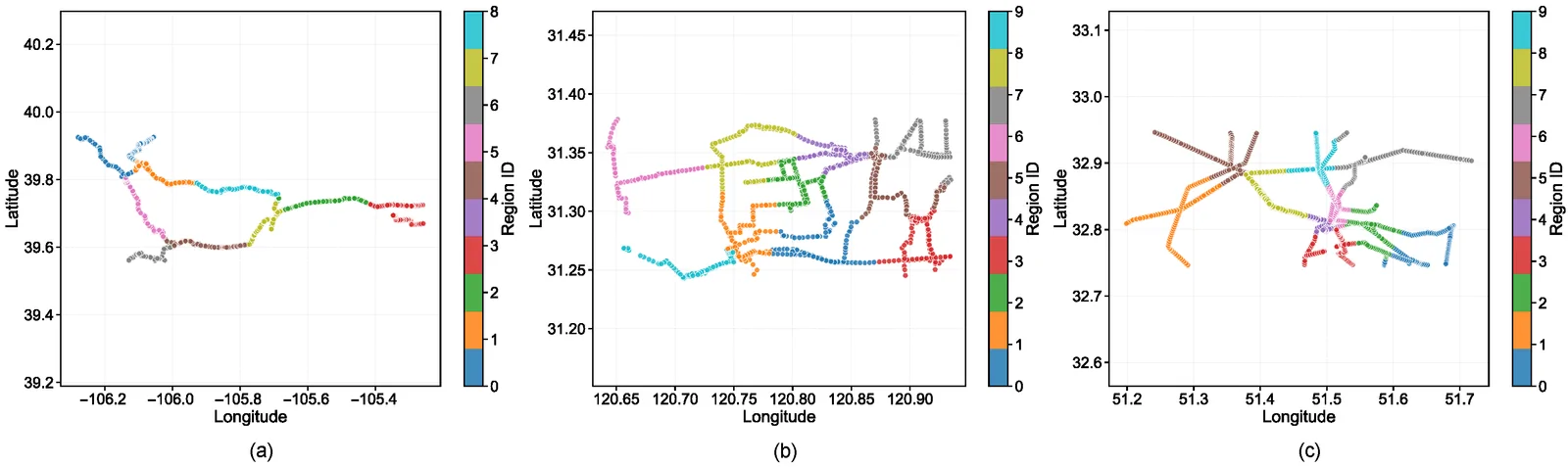
Visualization of spatial partitioning results by the DAR-DBSC algorithm under heterogeneous grid topologies. (**a**) Sparse chain-like corridors in the mountain scenario. (**b**) Complex mesh topology in the urban scenario. (**c**) Significant spatial density gradients in the energy hub scenario.

To intuitively assess the spatial coherence of the clustered structures, Figure 1 visualizes the spatial partitioning generated by DAR-DBSC across heterogeneous environments. The proposed algorithm reliably generates valid partitions across various transmission network scenarios. For the complex urban mesh topology characterized by high node density (Figure 1b), the algorithm decouples the global network into spatially compact local inspection blocks.

In the sparse, chain-like corridor scenario distributed along mountain ridges (Figure 1a), DAR-DBSC overcomes the “cutting-through effect” frequently induced by conventional Euclidean distance metrics. It preserves the chain-like local spatial connectivity encoded by the constructed affinity graph while satisfying the workload balancing constraints across all sub-regions.

Furthermore, in the energy hub scenario exhibiting sharp spatial density gradients (Figure 1c), the load-balanced clusters generated by the algorithm exhibit strong spatial adaptability. They form a compact and concentrated distribution within the central substation hub area, while smoothly extending along the peripheral sparse corridors. These visualization results indicate that, under strict physical and geographical constraints, DAR-DBSC yields a robust spatial partitioning scheme that simultaneously maintains local spatial affinity structure and improves multi-UAV workload balance.

Because the real-world data do not include complete transmission-line connectivity, topology-related results are interpreted with respect to the constructed spatial affinity graph rather than the physical grid graph.

### 4.3. Benchmark Evaluation of Trajectory Generation

Table 4 benchmarks the SSGC-PSEA architecture against LKH-3 [46] (a high-quality reference heuristic) and state-of-the-art NCO solvers (PPO [47], POMO [39], Sym-NCO [21], and AM-XL [48]). The proposed model achieves an optimal trade-off between solution accuracy and inference efficiency. Even with highly restricted domain-adaptation samples, it maintains robust cross-domain generalization for online deployment.

For a method with average route cost $C_{\mathrm{method}}$, the LKH-3-relative gap is defined as

(16) $${\mathrm{Gap}}_{\mathrm{LKH}-3}\left(\%\right)=\frac{C_{\mathrm{method}}-C_{\mathrm{LKH}-3}}{C_{\mathrm{LKH}-3}}\times 100.$$

This metric measures the relative difference from the LKH-3 reference solution and does not represent a certified global optimality gap.

For UAVs with constrained onboard resources, greedy decoding performance dictates deployment feasibility. Under greedy decoding, the proposed architecture exhibits strong adaptability to unseen topologies, yielding an LKH-3-relative gap of 1.8112%. Compared to constructive baselines like POMO (5.3541%) and Sym-NCO (5.4401%), the LKH-3-relative gap is reduced by approximately 66%. This superiority stems from overcoming the representational limitations of conventional models, which typically overfit absolute coordinate distributions and struggle with non-uniform tower topologies. Conversely, SSGC-PSEA implicitly embeds topologically isomorphic geometric priors into the network weights via the SSGC constraint. Consequently, it extracts manifold features of the underlying spatial layouts through a single forward pass, circumventing computationally expensive post-processing search.

With parallel instance augmentation (Augment ×8), the LKH-3-relative gap further decreases to 0.7528%, approaching the LKH-3 reference solution. Crucially, this high-quality performance relative to LKH-3 is achieved despite extremely scarce fine-tuning samples. Since acquiring annotated physical grid data is cost-prohibitive, expanding datasets to force a zero gap is impractical. These results demonstrate strong solving performance of the proposed method under limited-data conditions.

Computationally, as the reference heuristic, LKH-3 has a relative gap of 0.00% by definition; its average inference time per sample (1.068 s) is approximately 42 times that of the proposed augmented architecture (0.025 s), rendering it unsuitable for real-time dynamic replanning. Ultimately, SSGC-PSEA delivers high-precision path planning while satisfying sub-second latency and low data-dependency constraints, showing potential for low-latency path planning in the considered two-dimensional setting.

### 4.4. Synergistic Performance and Trajectory Visualization

**Figure 2:**
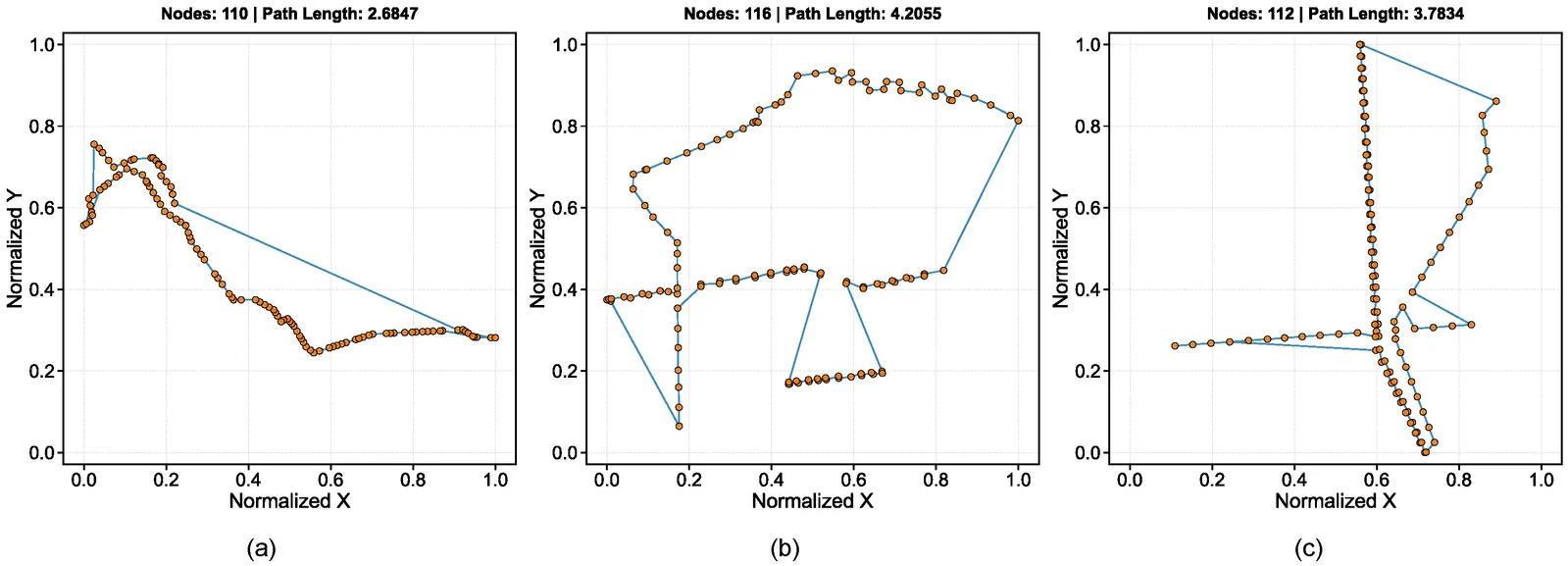
Visualization of optimal single-UAV inspection trajectories generated by the SSGC-PSEA algorithm within three typical heterogeneous sub-regions. (**a**) Sparse chain-like corridor in the mountain scenario. (**b**) Complex mesh topology with significant spatial voids in the urban scenario. (**c**) Non-uniform distribution featuring extreme density gradients in the energy hub scenario.

Figure 2 visualizes the optimal two-dimensional inspection trajectories across three heterogeneous scenarios, demonstrating the synergistic performance of the front-end DAR-DBSC spatial partitioning and the back-end SSGC-PSEA path optimization.

Avoiding trajectory “self-intersection” under Euclidean constraints serves as a practical standard for evaluating neural solvers. The trajectories generated by SSGC-PSEA maintain short total flight distances and exhibit smooth, intersection-free geometric characteristics. For the narrow mountain corridor scenario (Figure 2a), the routing follows a clear chain-like sequence. A single external long connection is utilized for the closed return trip, explicitly preventing redundant overlaps within local node clusters. The urban mesh scenario (Figure 2b) presents transmission networks severely fragmented by man-made infrastructures, creating non-uniform topologies with significant spatial voids. Navigating these complex structures typically induces diagonal misconnections; the proposed model, however, overcomes local optima to adaptively plan regular stair-step traversal paths along peripheral contours.

The energy hub scenario (Figure 2c) features a highly non-uniform node distribution with dense cores and sparse peripheries. Conventional constructive algorithms frequently suffer from trajectory “knotting” in such regions. The SSGC-PSEA algorithm forms a clear outer envelope and achieves parallel hairpin routing within high-density radial backbone corridors. Outbound and inbound connections remain strictly parallel and intersection-free despite tight spacing constraints.

These visualizations substantiate the engineering practicality of the proposed two-stage architecture. The front-end algorithm effectively decouples the scale of complex power grids via physical constraints. Grounded in high-order manifold representations, the back-end neural solver robustly adapts to extreme spatial distributions in real physical environments (e.g., narrow corridors, spatial voids, and sharp density gradients). The complete pipeline generates high-precision, end-to-end trajectories that demonstrate the potential for efficient collaborative inspection planning of the proposed method under the considered static two-dimensional setting.

## 5. Ablation Study

In this section, ablation studies, along with parameter sensitivity and convergence analyses, are incorporated to verify the efficacy of the core mechanisms and the robustness of the model against hyperparameter perturbations.

### 5.1. Ablation Study of the DAR-DBSC Algorithm

To evaluate the independent contributions of each core module, three ablation models are constructed. Model 1 (w/o GeoScaler) employs standard Z-score normalization instead of the underlying physical projection to analyze the impact of geometric fidelity. Model 2 (w/o Adaptive Graph) replaces the local density-aware connections with a globally fixed distance parameter to assess local spatial-affinity adaptation. Model 3 (w/o Feedback) removes the dynamic weight adjustment, maintaining a constant regularization parameter (γ) during optimization. For fair comparison, all variants are evaluated under identical random seeds and hyperparameters.

**Figure 3:**
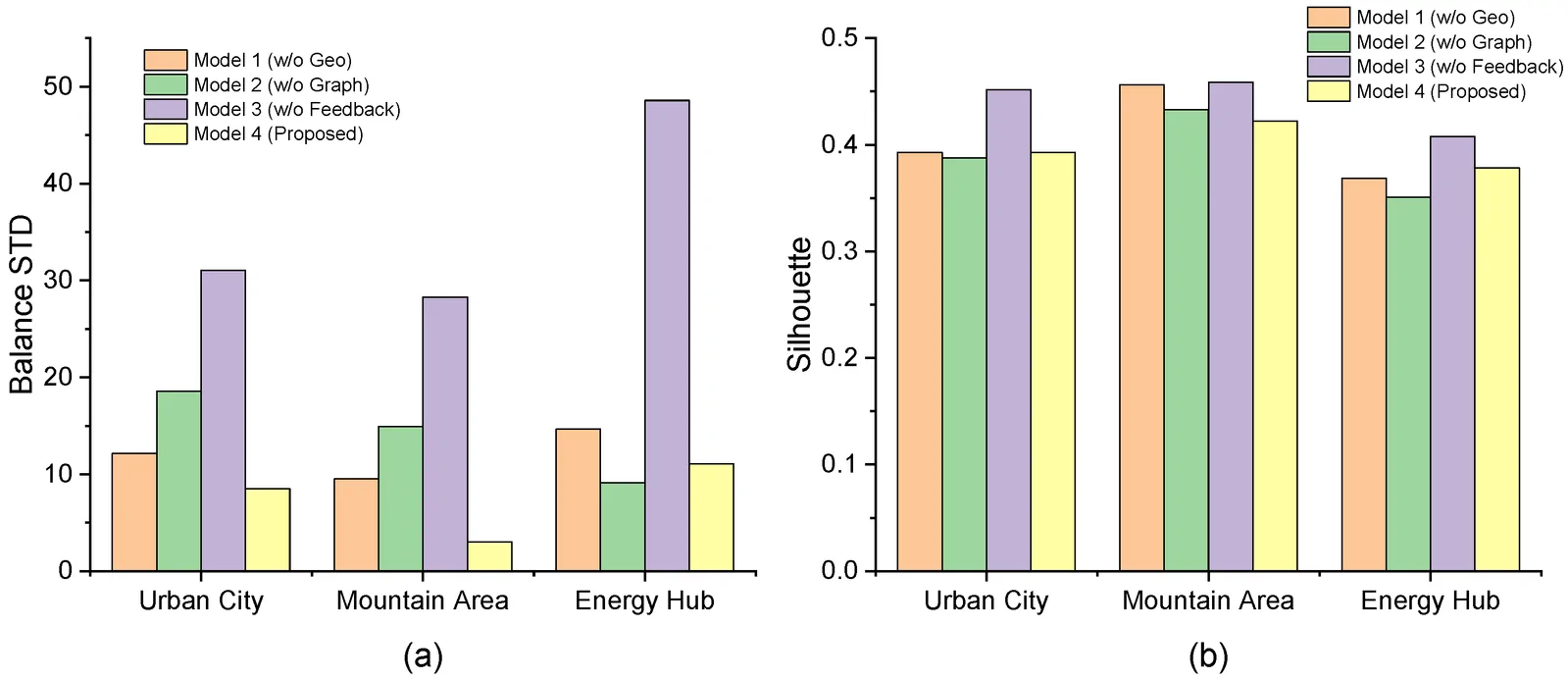
Quantitative comparison of the ablation study for the DAR-DBSC algorithm’s core modules across different grid scenarios. (**a**) Balance standard deviation ($\sigma_{B}$). (**b**) Silhouette coefficient (*S*).

Figure 3 illustrates the quantitative results. Removing the dynamic feedback (Model 3) severely degrades load balancing. In the energy hub scenario with sharp density gradients, the $\sigma_{B}$ of Model 3 spikes to approximately 50, performing worse than the standard DBSC baseline ($\sigma_{B}\approx 29$). This implies that while the adaptive graph captures spatial density gradients, a globally fixed regularization fails to suppress the strong spectral connectivity within dense hubs, leading to localized “giant clusters.” This validates the synergy between adaptive topology and dynamic feedback: the former extracts non-uniform physical features, while the latter adaptively reduces workload imbalance. Ultimately, the complete architecture substantially reduces $\sigma_{B}$ with only a marginal compromise in the Silhouette Coefficient (*S*) (Figure 3b), satisfying practical constraints for multi-agent collaborative task balancing.

### 5.2. Ablation Study of the SSGC-PSEA Algorithm

**Figure 4:**
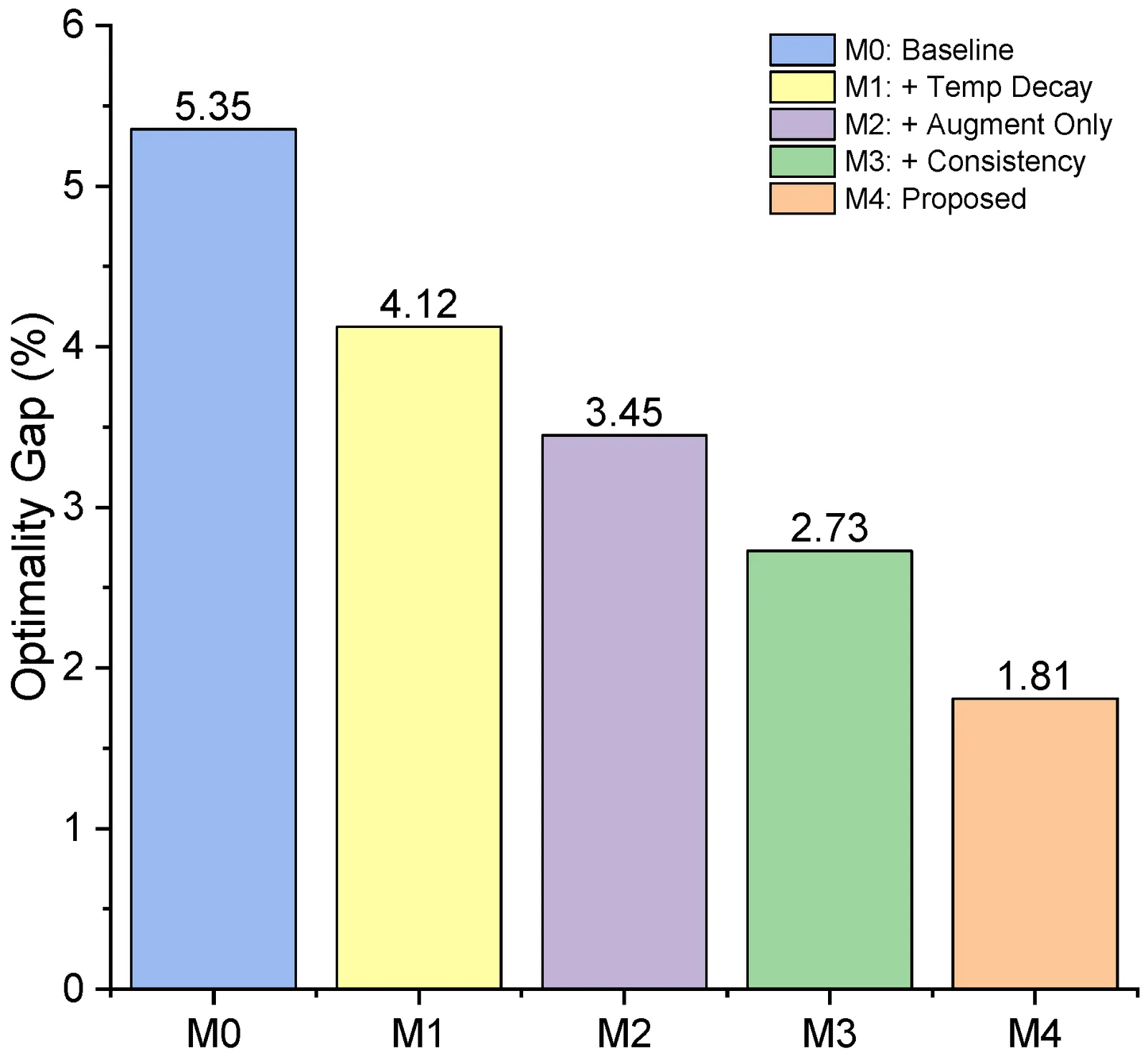
Ablation study of the SSGC-PSEA architecture. Comparison of the LKH-3-relative gap across different model variants.

To evaluate the core mechanisms of SSGC-PSEA, five ablation configurations are compared (Figure 4). M0 (Baseline POMO) yields a 5.35% LKH-3-relative gap, reflecting the susceptibility of conventional models to local optima in non-uniform topologies. Integrating PSEA (M1) reduces the gap to 4.12% by smoothing training gradients and suppressing step-wise numerical oscillations. To isolate data augmentation from consistency regularization, M2 introduces spatial symmetric inputs without the consistency loss, bottlenecking at a 3.45% gap. Incorporating the explicit SSGC loss (M3) further reduces the gap to 2.73%. This 0.72% improvement confirms that SSGC functions as an explicit manifold regularization mechanism rather than mere implicit data augmentation, guiding the network to extract geometrically invariant features. M4 combines PSEA, spatial-symmetry augmentation, and the SSGC consistency loss; compared with M3, it additionally introduces PSEA and achieves the lowest gap of 1.81%. This non-linear synergistic gain demonstrates that PSEA’s smooth parameter updates provide a stable environment for SSGC to extract high-order geometric features, guiding the policy toward high-quality solutions with improved generalization.

## 6. Discussion

### 6.1. Parameter Sensitivity and Convergence Analysis of DAR-DBSC

#### 6.1.1. Parameter Sensitivity Analysis

**Figure 5:**
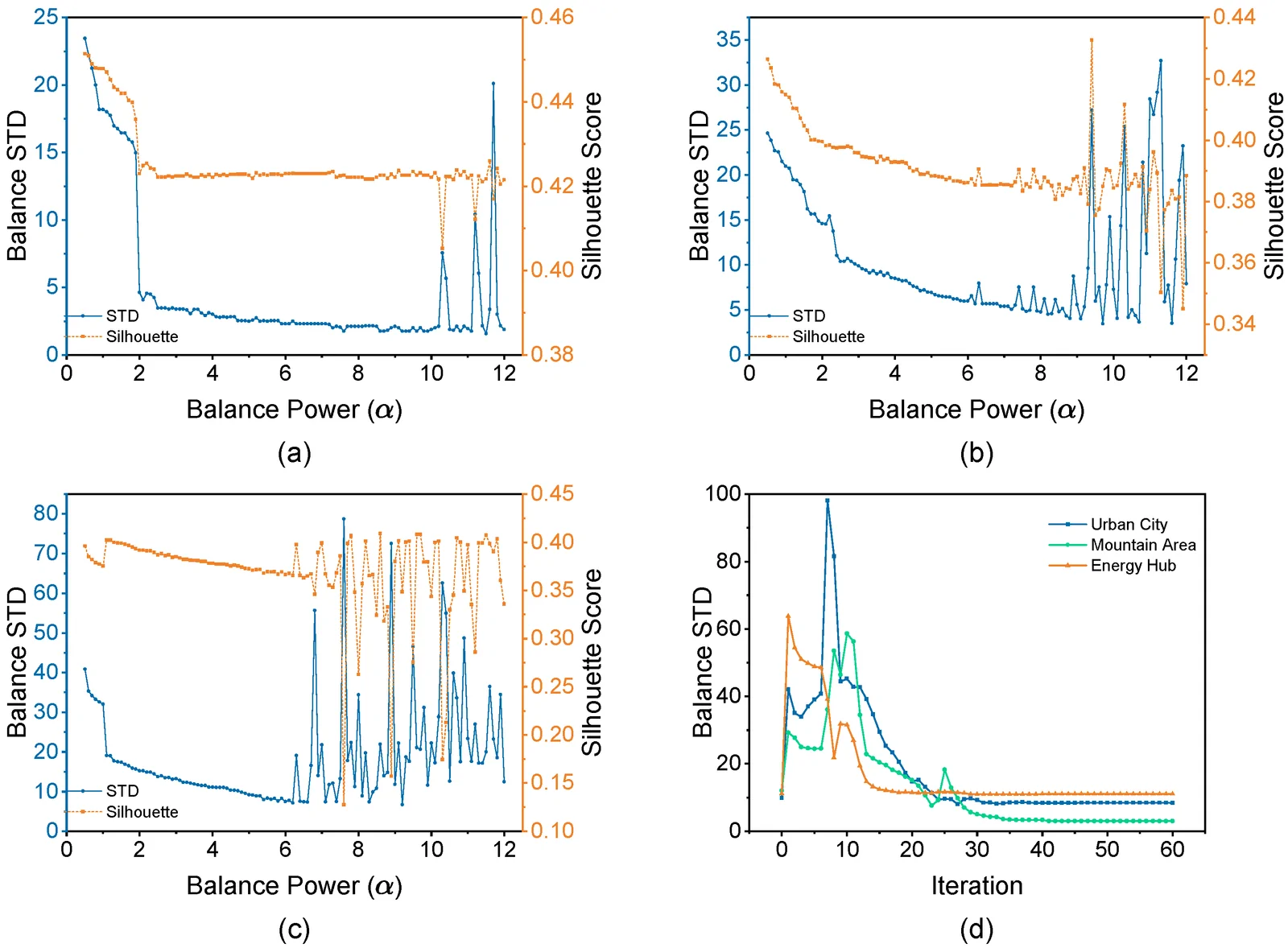
Parameter sensitivity and convergence analysis of the DAR-DBSC algorithm. Sensitivity of the balance power exponent α in the (**a**) mountain area, (**b**) urban city, and (**c**) energy hub scenarios. (**d**) Convergence curves of the balance standard deviation ($\sigma_{B}$) over iterations across different scenarios.

Figure 5 illustrates the impact of the balance power exponent (α) on clustering balance and local spatial affinity structure. Across all three typical scenarios, the balance standard deviation ($\sigma_{B}$, solid blue line) exhibits a consistent “decline–stabilize–oscillate” trend as α increases. For α<1.0, insufficient feedback fails to correct initial spatial skewness, keeping $\sigma_{B}$ high. As α enters the [2.0, 6.0] interval, $\sigma_{B}$ rapidly converges and stabilizes, while the Silhouette Coefficient (*S*, dashed orange line) experiences only a marginal decline. This confirms that the algorithm improves workload balance while retaining local spatial affinity structure within this optimal range.

Conversely, exceeding scenario-specific thresholds (e.g., α>6.5 in energy hubs, α>10.0 in mountain corridors) triggers severe metric oscillations. Excessive penalty weights disrupt the manifold constraints during spectral embedding, causing erratic partitioning transitions. Thus, α∈[ 2.0,6.0] is recommended for a robust trade-off between workload balancing and geometric fidelity.

#### 6.1.2. Convergence Dynamics Analysis

Figure 5d depicts the iterative evolution of $\sigma_{B}$. Unlike the smooth monotonic descent typical of traditional gradient methods, DAR-DBSC exhibits distinct non-monotonic convergence behaviors. During the initial phase (approximately the first 10 iterations), $\sigma_{B}$ displays transient oscillations with significant peaks (e.g., spiking to nearly 100 at iteration 8 in the urban scenario).

This initial spiking reflects the active intervention of the dynamic feedback mechanism: confronted with severe initial load skewness, the system executes a global graph reweighting. This breaks locally optimal partitions governed solely by geometric distances, triggering a short-term reorganization of task assignments. Following this spatial-affinity reconstruction, $\sigma_{B}$ drops rapidly across all scenarios, converging to a stable, low value by the 30th iteration. These trajectories empirically indicate stable optimization behavior after the initial exploration phase.

In this study, the number of inspection nodes is used as a workload proxy because each node is assumed to require an identical inspection operation. Route length, flight time, and energy consumption may also affect the actual UAV workload and will be incorporated into future joint task-allocation and routing models.

### 6.2. Robustness and Generalization Analysis of SSGC-PSEA

#### 6.2.1. Geometric Robustness Against Spatial Transformations

Evaluating generalization under unseen geometric transformations is essential to verify the extraction of underlying topological features. Two sets of experiments—multi-directional spatial flipping and cross-scale scaling—quantitatively assess how the SSGC-PSEA architecture mitigates anisotropic biases in Euclidean space.

**Figure 6:**
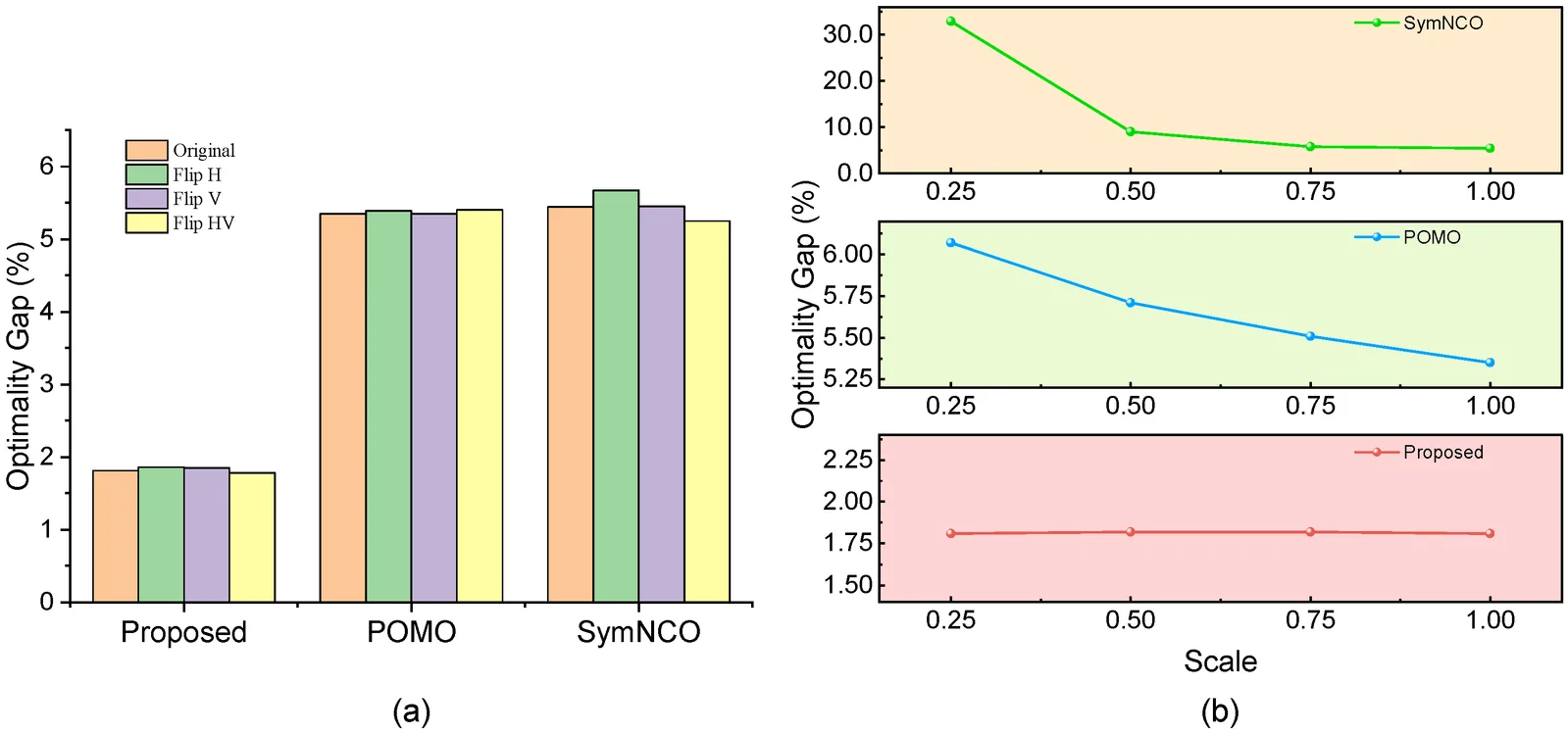
Generalization performance comparison of path planning models under multi-directional spatial flipping and cross-scale scaling conditions. (**a**) Bar chart of the LKH-3-relative gap under spatial flipping operations. (**b**) Line chart of the LKH-3-relative gap evolution under cross-scale scaling.

Figure 6a compares algorithmic robustness under discrete spatial mappings: original, horizontal flip (Flip H), vertical flip (Flip V), and hybrid flip (Flip HV). Under these quadrant transformations, the LKH-3-relative gaps of Standard POMO and Sym-NCO exhibit noticeable deviations and degradation. Conversely, the proposed method remains highly stable (maintained between 1.81% and 1.88%), indicating that its internal attention allocation is strictly independent of physical coordinate quadrant definitions.

Although uniform scaling preserves the TSP visiting order, it shifts the numerical distribution of input coordinates and distances; all neural solvers receive the same scaled inputs, and LKH-3 is re-executed at each scale. Figure 6b tracks performance as the testing graph scaling factor decreases from 1.00 to 0.25. As the underlying absolute distance metric shrinks, baseline algorithms exhibit significant instability: the gap of Standard POMO exceeds 6.00%, while Sym-NCO degrades sharply at a scale of 0.25, exceeding a 30.0% error. This failure stems from conventional solvers heavily relying on absolute distance distributions established during training. In contrast, the SSGC-PSEA error curve remains flat throughout the scaling interval. This result indicates improved robustness to coordinate-scale distribution shifts and reduced reliance on the numerical scale observed during training.

Ultimately, the SSGC mechanism improves robustness to directional, quadrant, and coordinate-scale variations. For wide-area UAV inspections, this provides profound engineering value: a single set of trained network weights can directly adapt to tasks with diverse spatial morphologies and spans (e.g., dense hubs to long-distance corridors). This eliminates the need for repeated retraining, drastically reducing deployment costs in complex field environments.

#### 6.2.2. Cross-Size Generalization of the Policy Model

Given that the number of nodes within operational sub-regions frequently varies in practical multi-UAV collaborative inspections, verifying zero-shot extrapolation to unseen network scales is imperative. To this end, a policy model pre-trained exclusively on 100-node instances (TSP-100) is evaluated on testing graphs ranging from 50 to 150 nodes. This setup strictly assesses the solver’s robustness against state-space expansion and contraction.

**Figure 7:**
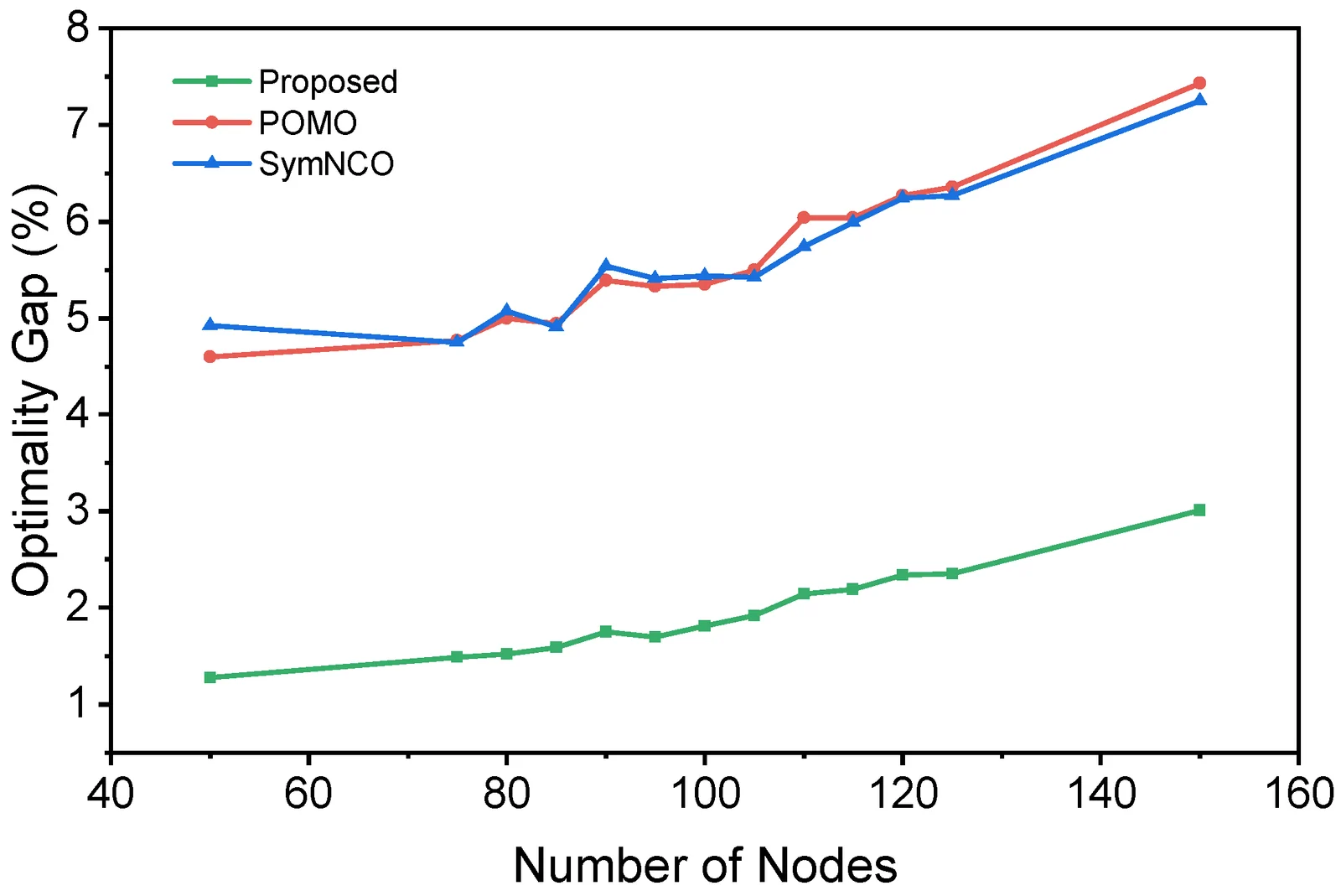
Evolution curves of the LKH-3-relative gap for various neural solvers under cross-size extrapolation testing (all models are pre-trained exclusively on 100-node instances).

Figure 7 illustrates the LKH-3-relative gap as the problem size scales from 50 to 150. Although all algorithms exhibit an upward trend, the baselines (POMO and Sym-NCO) suffer significant degradation within the extrapolation interval (>100 nodes), with errors exceeding 7.2% at 150 nodes. This vulnerability is fundamentally rooted in the inherent limitations of conventional attention mechanisms: during training, they overfit fixed node cardinalities and absolute coordinate distributions. As a result, when the testing scale expands and alters global relative distance spans, the original attention allocation struggles to maintain effective long-range feature extraction.

By comparison, SSGC-PSEA demonstrates robust cross-size adaptability with a remarkably flat error evolution curve. Even when extrapolating to 150 nodes, the optimization error is contained at approximately 3.0% (an approximate 58% reduction compared to baselines). This compelling evidence substantiates that the SSGC constraint successfully guides the network to extract geometrically invariant relative manifold features rather than overfitting to fixed node cardinalities or global absolute coordinates. Such high-quality manifold representations fundamentally decouple the path planning logic from the specific scale of the input graph.

Accordingly, the proposed model generalizes local optimization rules learned at the 100-node scale to complex sub-regions of varying sizes. This zero-shot generalization capability eliminates the need for repeated retraining across heterogeneous networks, supporting deployment under the evaluated static planning settings.

## 7. Conclusions

This paper proposes a two-stage hierarchical planning framework to address the key challenges of spatial partitioning and trajectory generation in multi-UAV collaborative inspections of heterogeneous power grids. The proposed architecture integrates the DAR-DBSC algorithm and the SSGC-PSEA neural solver to support efficient and load-balanced operations. Extensive experiments on real-world grid datasets across diverse scenarios demonstrate clear improvements over state-of-the-art baselines. Specifically, DAR-DBSC effectively minimizes workload imbalance while preserving local spatial affinity structure. Meanwhile, SSGC-PSEA lowers the LKH-3-relative gap to 1.81% under greedy decoding and exhibits strong spatial geometric robustness and cross-size generalization. Moreover, ablation studies highlight the necessity and complementarity of the dynamic feedback, geometric consistency, and entropy annealing modules. The results suggest that this framework provides a robust and scalable solution for multi-UAV inspection under data-scarce conditions. The current study adopts a static two-dimensional routing abstraction and does not explicitly model operational factors such as energy limits, flight dynamics, obstacles, no-fly zones, weather, communication, or inter-UAV separation. Accordingly, the present results should be interpreted as evidence of static planning performance rather than validation of direct real-world deployment. Although the proposed method performs well in static grid topologies, adapting to dynamic constraints and real-time node failures presents opportunities for further improvement. In future work, decentralized online replanning and distributed communication protocols should be further studied to improve the system’s resilience and adaptivity in highly dynamic physical environments.

## Figures and Tables

**Table 1:** Common Hyperparameters Used in DAR-DBSC.

Hyperparameters	Values
number of clusters *c*	[N/100]
number of anchors *m*	min (300,N)
nearest neighbors *k*	30
initial regularization $\gamma_{\mathrm{base}}$	0.05
balance power α	4
max outer iterations $T_{\max}$	60
inner GPI iterations $T_{\mathrm{GPI}}$	10
feedback start epoch $T_{\mathrm{start}}$	5

**Table 2:** Common Hyperparameters Used in SSGC-PSEA.

Hyperparameters	Values
number of nodes *N*	100
number of epochs	100
fine-tuning epochs	10
batch size	64
learning rate	$1\times 10^{-4}$
fine-tuning learning rate	$1\times 10^{-5}$
lr decay milestones	[80, 95]
synthetic pre-training instances	160,000
real-world fine-tuning instances	20,000
real-world test instances	300 (100 per scenario)
consistency weight β	0.5
initial temperature $\tau_{max}$	1.5
min temperature $\tau_{min}$	0.1

**Table 3:** Performance Comparison of Different Spatial Partitioning Algorithms Across Three Grid Scenarios.

Scenario	Method	Balance STD ($\sigma_{B}$) ↓	Load Range ($R_{L}$) ↓	Silhouette (*S*) ↑	Runtime (*T*)/s
Urban City (Complex Mesh)	K-Means	29.86	89	0.4546	0.2514
	Spectral Clustering	40.15	151	0.3030	0.4919
	DBSC	29.76	92	0.4484	1.0674
	DAR-DBSC	8.38	26	0.3922	1.1886
Mountain Area (Linear Corridor)	K-Means	30.49	105	0.5072	0.2123
	Spectral Clustering	38.01	113	0.4000	0.3950
	DBSC	33.02	111	0.4732	0.9194
	DAR-DBSC	3.02	9	0.4221	1.1759
Energy Hub (Density Gradient)	K-Means	60.99	232	0.4432	0.2071
	Spectral Clustering	52.25	144	0.3546	0.3916
	DBSC	29.07	116	0.4264	1.0802
	DAR-DBSC	11.09	40	0.3783	1.2448

**Table 4:** Benchmark Performance Comparison of Different Methods on the Real-World Transmission Tower TSP-100 Dataset.

Method	Inference Strategy	Time per Sample (s)	Cost ↓	LKH-3-Relative Gap (% ↓)
LKH-3	-	1.068	3.97728	0.00 (reference)
Single-Trajectory				
AM-XL	Greedy	0.00726	4.69201	17.9704
PPO	Greedy	0.00698	5.57993	40.2950
Standard POMO	Greedy	0.00888	4.19023	5.3541
Sym-NCO	Greedy	0.00929	4.19365	5.4401
Proposed	Greedy	0.00892	4.04932	1.8112
Multi-Trajectory				
Standard POMO	Augment ×8	0.02462	4.06402	2.1809
Sym-NCO	Augment (Aug ×8)	0.02209	4.06427	2.1872
Proposed	Augment (Aug ×8)	0.02506	4.00722	0.7528
Stochastic Sampling				
Standard POMO	Sampling (N=100)	0.01008	4.23526	6.4863
Proposed	Sampling (N=100)	0.01020	4.06191	2.1278

## Data Availability

The data that support the findings of this study are available upon request from the corresponding author. The data are not publicly available due to privacy and confidentiality restrictions related to critical power grid infrastructure.
